# Anandamide Revisited: How Cholesterol and Ceramides Control Receptor-Dependent and Receptor-Independent Signal Transmission Pathways of a Lipid Neurotransmitter

**DOI:** 10.3390/biom8020031

**Published:** 2018-05-22

**Authors:** Coralie Di Scala, Jacques Fantini, Nouara Yahi, Francisco J. Barrantes, Henri Chahinian

**Affiliations:** 1 INMED, INSERM U1249, Parc Scientifique de Luminy, 163 Avenue de Luminy, BP13 13273 Marseille CEDEX 09, France; coralie.discala@gmail.com; 2 INSERM UMR_S 1072, Aix-Marseille Université, 13015 Marseille, France; jm.fantini@gmail.com (J.F.); nouara.y@gmail.com (N.Y.); 3 Laboratory of Molecular Neurobiology, Biomedical Research Institute (BIOMED), UCA–CONICET, Av. Alicia Moreau de Justo 1600, C1107AFF Buenos Aires, Argentina; rtfjb1@gmail.com

**Keywords:** endocannabinoid, lipid raft, cholesterol, ceramide, sphingolipid, cannabinoid receptor, molecular docking, protein-lipid interaction

## Abstract

Anandamide is a lipid neurotransmitter derived from arachidonic acid, a polyunsaturated fatty acid. The chemical differences between anandamide and arachidonic acid result in a slightly enhanced solubility in water and absence of an ionisable group for the neurotransmitter compared with the fatty acid. In this review, we first analyze the conformational flexibility of anandamide in aqueous and membrane phases. We next study the interaction of the neurotransmitter with membrane lipids and discuss the molecular basis of the unexpected selectivity of anandamide for cholesterol and ceramide from among other membrane lipids. We show that cholesterol behaves as a binding partner for anandamide, and that following an initial interaction mediated by the establishment of a hydrogen bond, anandamide is attracted towards the membrane interior, where it forms a molecular complex with cholesterol after a functional conformation adaptation to the apolar membrane milieu. The complex is then directed to the anandamide cannabinoid receptor (CB1) which displays a high affinity binding pocket for anandamide. We propose that cholesterol may regulate the entry and exit of anandamide in and out of CB1 by interacting with low affinity cholesterol recognition sites (CARC and CRAC) located in transmembrane helices. The mirror topology of cholesterol binding sites in the seventh transmembrane domain is consistent with the delivery, extraction and flip-flop of anandamide through a coordinated cholesterol-dependent mechanism. The binding of anandamide to ceramide illustrates another key function of membrane lipids which may occur independently of protein receptors. Interestingly, ceramide forms a tight complex with anandamide which blocks the degradation pathway of both lipids and could be exploited for anti-cancer therapies.

## 1. Introduction

Chemical neurotransmission is a discontinuous and cyclic phenomenon controlled by the neurotransmitter levels in the inter-neuronal space—the synaptic cleft—separating pre- and post-synaptic neurons. Several mechanisms of neurotransmission can be described according to the type of neuron and its associated neurotransmitter(s). In general, neurotransmitter levels are specifically regulated by transporter-dependent reabsorption mechanisms located in the membrane of the presynaptic neuron or, in some cases, of glial cells. Historically, glia has been considered as an accessory cellular compartment helping neuronal cells in their “noble” functions, chiefly nerve impulse transmission and synaptic communication. Over the last ten years, the concept of a tripartite synapse including the contribution of astrocytes to the classical pre- and post-synaptic neuronal duet has gradually improved our knowledge in this area. In a few cases, the neurotransmitter is deactivated in the synaptic cleft by a specific enzyme-dependent hydrolytic process located at the post-synaptic neuronal membrane, as is the case for the ubiquitous neurotransmitter acetylcholine. However, as noted by Südhof, our comprehension of brain function is strongly limited by the fact “that most of its molecular processes are incompletely understood” [1]. Among these gaps in knowledge, one could cite the relative lack of studies focused on the physico-chemical properties of neurotransmitter molecules, and especially their behavior in water. In the case of lipid neurotransmitters such as endocannabinoids, it is of primary importance to decipher the molecular mechanisms controlling their transfer from the aqueous phase of the synaptic cleft to the plasma membrane of the receptive neuron. The aim of this review is to present an overview of these mechanisms and to identify new potential regulatory functions for cholesterol and ceramides in the endocannabinoid system. To enrich the discussion, some relevant unpublished data obtained by Di Scala, Yahi, Fantini, and Chahinian are also presented and discussed. 

## 2. Membrane Domains and the Synapse: General Aspects

The plasma membrane is now generally considered to be a mosaic of cholesterol-enriched lipid domains bathing in a more liquid phase enriched in phosphatidylcholine [2]. The concept of lipid rafts, proposed in 1997 by K. Simons and E. Ikonen [3], has been the subject of intense discussion and controversy, although a consensual definition was adopted during the 2006 Keystone Symposium on Lipid Rafts and Cell Function [4]. In the review stemming from this symposium, lipid rafts are defined as “small (10–200 nm), heterogeneous, highly dynamic, sterol-and sphingolipid-enriched domains that compartmentalize cellular processes” [4]. Despite this consensual statement, several issues remain open, especially since there are data suggesting that lipid raft domains are the major component of the plasma membrane [5,6]. 

The distribution of cholesterol between non-raft and raft domains remains controversial [7,8] Our discussion will focus on the molecular interactions between anandamide and membrane lipids and/or between anandamide and its receptors. We will also consider the physical phase in which these interactions take place, assuming that lipid rafts corresponded more to the more condensed liquid-ordered (Lo) areas than the surrounding liquid-disordered (Ld) matrix, a postulation in accordance with the modern views of the plasma membrane [2,6,9]. As we will see, synaptic transmission cannot be fully explained without considering the lipids present in the post-synaptic membrane, including those that surround neurotransmitter receptors [9,10]. Among these lipids, gangliosides and cholesterol play prominent roles and are able to interact with both the neurotransmitter and its receptors in the post-synaptic membrane. There is a plethora of scientific literature devoted to the localization of neurotransmitter receptors (both ionotropic and metabotropic receptors) within or outside lipid rafts (most often within) [11,12]. In contrast, the interaction of neurotransmitters with post-synaptic membrane lipids has received scant appraisal. In the case of anandamide, the problem is particularly complex due to the lipid nature of the neurotransmitter itself. As explained in this review, the anandamide synapse is controlled by a series of water–lipid, lipid–lipid and lipid–protein interactions that occur in various environments. Taking into account this ballet of molecular interactions led us to reconsider the anandamide synapse as a membrane-dependent physico-chemical process and more generally to revisit the biological functions of anandamide at the molecular level. 

## 3. Biochemical and Physico-Chemical Singularities of the Anandamide Synapse

**Figure 1:**
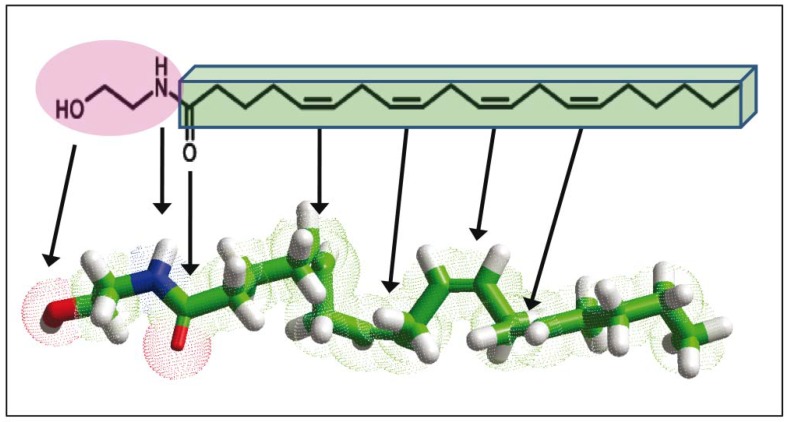
Chemical structure of anandamide (AEA). Anandamide is derived from arachidonic acid. The apolar moiety (in the box) contains 4 double bonds. The polar part (in mauve) is an ethylamine group. In the molecular model carbon atoms are in green, nitrogen in blue, oxygen in red, and hydrogen in white.

Anandamide, also known as *N*-arachidonoylethanolamine (AEA), is a neurotransmitter belonging to the endocannabinoid family [13]. Like other brain lipids collectively referred to as bioactive lipids [14], AEA has distinctive signaling functions [15], among which are pharmacological effects similar to those of Δ9-tetrahydrocannabinol, the psychoactive component of marijuana [16]. It is biosynthesized from arachidonoyl acid- containing membrane glycerophospholipids in areas of the brain functionally related to higher cognitive processes, motivation, and movement control [17,18]. The chemical structure of anandamide is shown in Figure 1. 

**Figure 2:**
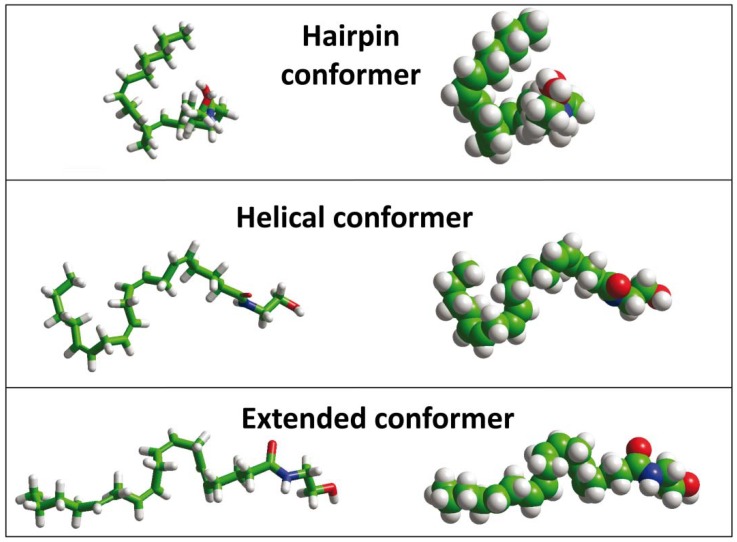
The three main types of AEA conformers. The conformers were generated through iterative rounds of molecular dynamics simulations with the chemistry at Harvard macromolecular mechanics (CHARMM) force field of the Hyperchem^TM^ professional program 8.0 (Hypercube, Inc., 1115 NW 4th Street, Gainesville, Florida 32601, USA) [23,24].

From a chemical point of view, AEA is a derivative of arachidonic acid, with which it shares the polyunsaturated hydrocarbon chain. The chemical modification of arachidonic acid that leads to the formation of anandamide consists in the addition of a neutral ethanolamine group. Arachidonic acid displays a carboxylic acid group that is dissociated into carboxylate and a proton, around neutral pH [19]. The pKa of this polyunsaturated fatty acid (i.e., the pH at which 50% of the fatty acid molecules are protonated and 50% deprotonated) has been estimated between 7–8 (depending on the concentration of arachidonic acid) by titration methods [20,21]. Therefore, at physiological pH, arachidonic is a mixture of protonated and deprotonated forms. The addition of ethanolamine into the arachidonic acid moiety generates a compound that no longer possesses an ionizable group (Figure 1). Moreover, anandamide displays one nitrogen and two oxygen atoms that can accept as many as five hydrogen bonds, together with two donor hydrogen atoms. Thus, the polar head group of anandamide can interact with a crown of water molecules via a total of seven hydrogen bonds. In contrast, arachidonic acid has two oxygen atoms and (for around 50% of the molecules) one hydrogen, so that its polar head group may form only five hydrogen bonds with surrounding water molecules. Thus, the chemical modification of arachidonic acid renders it slightly more soluble in water due to the highest possible number of hydrogen bonds. The apolar chain of arachidonic acid and anandamide is also singular in that it displays a series of four double bonds with the *cis* (*Z*) configuration. Each double bond induces a kink in the axis of the hydrocarbon chain of about 30°. With four double bonds, the successive kinks favor a hairpin-like shape of the chain (120°) which can thus adopt a compact structure that markedly differs from the classical topology of a long hydrocarbon chain immersed in a biological membrane [22]. An example of an energy-minimized hairpin conformer of anandamide is shown in Figure 2. 

The flexibility of polyunsaturated hydrocarbon chains is also compatible with larger structures corresponding to the helical and extended conformers illustrated in Figure 2. Taken together, we can conclude that anandamide combines two distinct features, one inherited from arachidonic acid (conformational diversity) and the other one from ethanolamine (improved solubility in water). Nevertheless, anandamide remains a lipid molecule and its solubility in water—also significantly enhanced when compared to arachidonic acid—is still very low when compared to more classical neurotransmitters such as glutamate or even serotonin. Therefore, the modalities of an anandamide synapse have to be specific for this type of lipid-derived neurotransmitter. In particular, anandamide might encounter severe difficulties in diffusing through the synaptic cleft and reaching its receptors. Another typical feature of anandamide-based neurotransmission is that, in contrast with the classical scheme of the chemical synapse, it may operate in the retrograde direction, that is, from the post- to the pre-synaptic neuron [25,26]. 

## 4. Anandamide Diffusion through the Synaptic Cleft: Alone or Accompanied?

**Figure 3:**
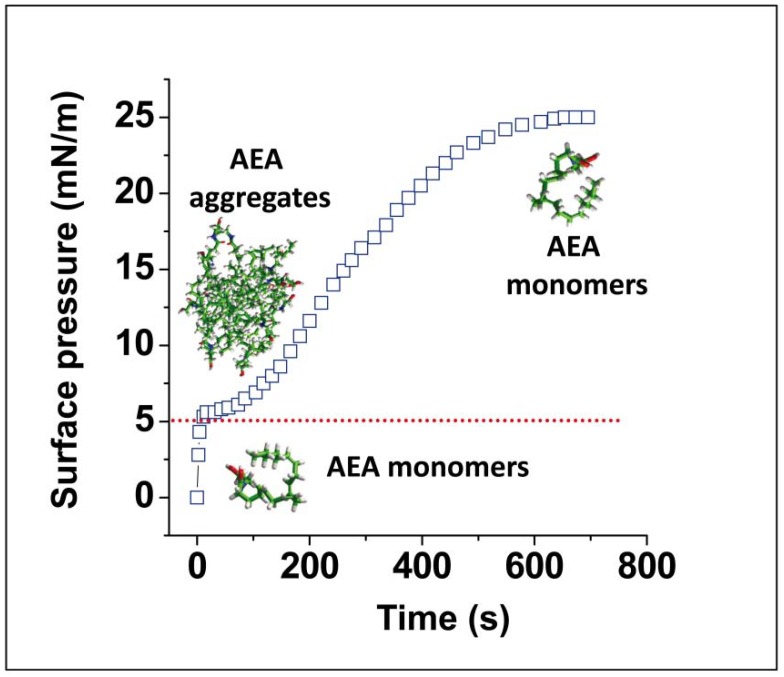
Surface activity of AEA monomers and aggregates. Anandamide is injected in pure water and its dissolution is assessed by surface pressure measurements using a Langmuir microtensiometer. Monomers immediately reach the air–water interface (first phase, sharp increase) whereas aggregates deliver AEA monomers very slowly (second phase, sigmoid curve) [28].

For the reasons explained above, AEA diffusion in the synaptic cleft may require a vehicle. This question has not been fully resolved. Several possibilities can be envisaged, such as a carrier protein, a lipid vesicle, or an exosome. From a physico-chemical point of view, and as can be illustrated by a simple experiment, the limit of AEA solubility in water is very low: when it is mixed with water, it does not create a clear solution. In fact, most AEA molecules aggregate into a precipitate that can be observed under an optical microscope [9] and only a small proportion of AEA is actually dissolved in water as monomers. Such monomers spontaneously migrate to the air–water interface and this phenomenon can be quantified by surface pressure measurements with a Langmuir microtensiometer [27]. As shown in Figure 3, when AEA is confronted with an aqueous phase, only 20% of the molecules can immediately reach the interface, which means that 80% of AEA molecules are clustered in large aggregates whose breakup occurs slowly and with latency. The progressive dissolution of the AEA aggregates can be quantitatively observed as a gradual increase in surface pressure after the first phase. 

**Figure 4:**
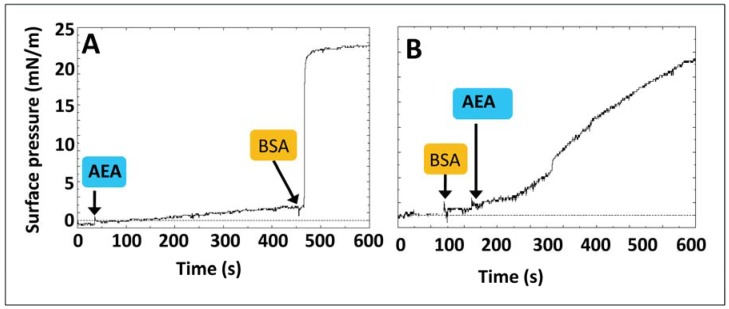
Protein-assisted dissolution of AEA. Low amounts of AEA (in the nanomolar range) do not contain sufficient amounts of AEA to trigger and thus do not significantly increase the surface pressure (**A**). The addition of lipid-free bovine serum albumin (BSA) induces the immediate dissolution of AEA aggregates (surface pressure increase). Bovine serum albumin by itself has no effect on the surface pressure (**B**). Under these conditions, the addition of AEA (the same concentration as in panel A) resulted in a sudden increase in surface pressure, indicating that BSA immediately dissolved AEA. The effects of BSA on AEA solubility were discussed in Chapter 5 of reference [9].

The sigmoidal curve of Figure 3 suggests that the process is cooperative and that it takes around 10 min to reach saturation. Moreover, it depends on the concentration of AEA, since lower amounts of the neurotransmitter do not significantly affect the value of the surface pressure, as shown in Figure 4A. 

Under these unfavorable conditions, the addition of nanomolar concentrations of bovine serum albumin (BSA), a well-known lipid binder and transporter, results in a sudden, rapid, and dramatic increase in the surface pressure. Since at the concentration of BSA used in this experiment, the protein has, by itself, no detectable effect on the surface pressure (Figure 4B), it can be concluded that BSA induces an instantaneous disaggregation of AEA. Taken together, these observations underscore the absolute requirement of a binder/transporter for the diffusion of a lipid in water. In the brain, this function can be fulfilled by various lipid-binding proteins including α-synuclein which can transport arachidonic acid [29], or by a lipid-based transport system such as exosomes [30]. Interestingly, surface pressure measurements have shown that BSA can be functionally replaced by α-synuclein, which displays a similar efficiency for dissolving AEA aggregates and is able to transport the monomers to the interface [9]. It is important to note that this effect on AEA is highly specific since non-relevant proteins such as immunoglobulin do not fulfill this function [9]. From a practical point of view, these data may raise questions about the current use of organic solvents such as dimethyl sulfoxide (DMSO) or ethanol to dissolve AEA for experimental use in neural systems (e.g., isolated cells or brain slices). Indeed, these organic solvents may affect the physical state [31,32] or even the lipid composition [33] of the plasma membrane and thus artifactually increase (or inhibit) the action of anandamide on G protein-coupled receptors (GPCRs). As alternative vehicles for AEA we suggest the use of BSA [19] or methyl-β-cyclodextrin (β-MCD), with the restriction that the latter should be used at concentrations that do not induce any lipid extraction from biological membranes [34].

## 5. Anandamide Insertion in the Plasma Membrane: A Cholesterol-Dependent Process

The main receptors for anandamide and other endocannabinoids (e.g., 2-arachidonoyl-*sn*-glycerol, 2-AG) belong to the family of GPCRs. Both central (CB1) and peripheral (CB2) receptors have been characterized [35]. Due to its lipid nature, it is unlikely that AEA could directly bind to an extracellular region of these receptors. Consistent with the lipid nature of AEA, its binding domain is located deep in the plasma membrane and has thus to be located within one or several transmembrane helices of the receptor [36,37]. However, before targeting the apolar parts of the receptor, AEA has first to penetrate the lipid bilayer, and then diffuse in the membrane until it reaches its receptor [37,38]. In theory, this process can be considered as either spontaneous (lateral diffusion through the lipid phase) or assisted by a membrane carrier. The existence of such AEA transporters, generally considered to be proteins, was first hypothesized some time ago [39,40,41] and has been the subject of an intense and still open debate [42,43]. On the basis of a series of physico-chemical experiments with reconstituted membrane systems, our group has identified cholesterol as a potential non-protein-mediated but particularly efficient AEA transporter [34]. Although this finding does not preclude the possibility of protein involvement in AEA transport, it demonstrates that cholesterol, by itself, is sufficient to fulfill this function.

Based on these considerations, we hypothesize that the insertion of AEA in the membrane is a cholesterol-dependent process. We have experimentally shown that AEA interacts specifically with cholesterol in reconstituted membrane models consisting of either monolayers or bilayers [34]. What are the molecular properties of cholesterol explaining its specific interaction with AEA? At first glance, it may be difficult to accept that a simple lipid molecule such as AEA could exhibit a particular specificity for cholesterol in a biological membrane containing myriads of other lipids. However, the interaction of two biomolecules depends on both their chemical compatibility and geometric shape. There are various examples of chemical compatibility, including electrostatic interactions between anionic and cationic groups, hydrogen bonds between donor and acceptor groups, and van der Waals forces. In terms of geometry, there are always constraints that may either allow or prevent the accessibility of chemically compatible groups. In the case of membrane lipids, additional parameters should be taken into account, in particular the melting temperature ensuring that both lipids are in the same physical state at physiological temperature [44]. In the case of AEA and cholesterol, this latter parameter is critical because, in the plasma membrane, cholesterol is found both in the Lo [45] and in the Ld phase [46]. However, the cholesterol molecules found in lipid rafts are tightly bound to sphingolipids and thus might not be easily accessible to AEA delivered to the membrane by a carrier protein. This particular issue is discussed in detail in the next section.

**Figure 5:**
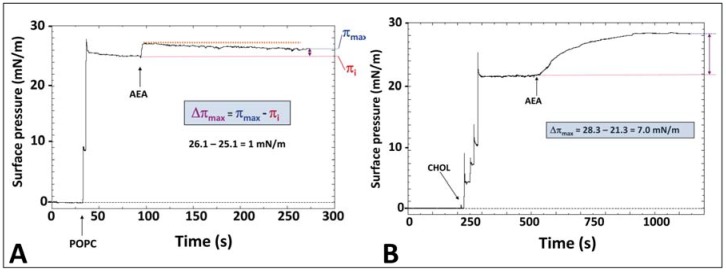
Anandamide interacts preferentially with cholesterol vs., phosphatidylcholine. (**A**) A monolayer of phosphatidylcholine (POPC) was prepared at the air–water interface at a stable value of 25.1 mN·m^−1^. Then AEA was injected in aqueous subphase. It slightly increased the surface pressure which rapidly returned to the initial value. After 5 min, the difference between the surface pressure after and before the addition of AEA (Δπmax) was as low as 1.0 mN·m^−1^ (no significant interaction). (**B**) A similar experiment as in panel (**A**) but with a monolayer of cholesterol. In this case, Δπmax was >7.0 mN·m^−1^(significant interaction). These data correspond to the untreated raw data used for Figure 3 in reference [34].

The specificity of the interaction between AEA and cholesterol can be demonstrated by surface pressure measurements of reconstituted membrane systems [47,48,49]. In these experiments, a lipid monolayer is prepared at the air–water interface and AEA is injected in the aqueous subphase underneath the monolayer. The insertion of AEA in the lipid monolayer is then quantified by real-time measurements of the surface pressure. An illustration of this method is given in Figure 5A (for a phosphatidylcholine monolayer) and in Figure 5B (for a cholesterol monolayer). 

The first step consists of the formation of the lipid monolayer which is performed by the addition of 1–2 µL of the lipid dissolved in chloroform/methanol. Each drop induces a distinct increase in the surface pressure which, after several additions, reaches the expected value determined by the operator. In the case of palmitoyl-oleoyl-phosphatidylcholine (POPC), a stable pressure of 25.1 mN·m^−1^ was obtained (Figure 5A). After several minutes (sufficient to allow the evaporation of residual solvent), AEA was injected in the subphase. The continuous recording of the surface pressure of the phoshatidylcholine monolayer indicated that only a few AEA molecules were able to penetrate this membrane. In contrast, when AEA was injected beneath a cholesterol monolayer, the surface pressure gradually increased to reach a stable plateau value of 7.0 mN·m^−1^ above the initial pressure (Figure 5B). Taken together, these physico-chemical data demonstrate that AEA has a marked preference for cholesterol vs. phosphatidylcholine. This type of experiment was performed with a series of membrane lipids [34,50]. From these data, we could conclude that AEA interacts with cholesterol or ceramide, but does not recognize other important membrane lipids such as sphingomyelin or glycosphingolipids.

**Figure 6:**
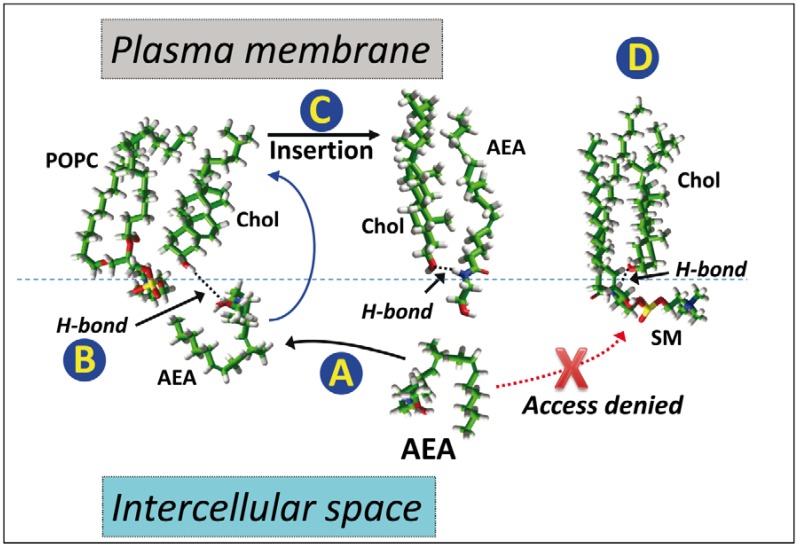
In silico studies of AEA insertion in a cholesterol-containing membrane. (**A**) When delivered by a transport protein (e.g., serum albumin or α-synuclein), AEA is still surrounded by water molecules and thus should adopt a compact, hairpin conformation. (**B**) It first interacts with the –OH group of cholesterol in the phosphatidylcholine (POPC)-rich liquid-disordered (Ld) phase. (**C**) Then, cholesterol triggers the insertion of AEA which acquires an extended conformation. The AEA/cholesterol complex is stabilized by both van der Waals forces and a hydrogen bond (H-bond). (**D**) Note that in the liquid-ordered (Lo) phase, cholesterol interacts with sphingolipids (e.g., sphingomyelin, SM) which deny access to AEA because they mask the polar head group of cholesterol (“umbrella” effect). Experimental data linked to these mechanisms have been published in reference [34].

Molecular dynamics simulations showed that there is both chemical compatibility and geometrical complementarity between AEA and cholesterol (Figure 6). 

The molecular mechanism of AEA/cholesterol interactions has revealed the critical role of a hydrogen bond between the –OH group of the sterol and the –NH group of AEA. This hydrogen bond can be formed as soon as AEA is delivered to the membrane by its carrier protein. At this stage, AEA is still in a hairpin conformation since the lipid has to face water molecules located between the membrane surface and the transport protein. Once this hydrogen bond is established, cholesterol triggers the insertion of AEA within the membrane via a flip-flop mechanism allowing the formation of van der Waals interactions that further stabilize the complex. The conformation of AEA now shifts from a hairpin to an extended shape, which underscores the importance of the conformational flexibility of anandamide for its biological activity. This insertion process is unlikely to occur within lipid rafts, where cholesterol is totally masked by sphingomyelin through an “umbrella” mechanism [51,52] stabilized by a hydrogen bond between the –OH group of cholesterol and the –NH group of sphingomyelin and is thus inaccessible to AEA. In contrast, the cholesterol molecules immersed in the phosphatidylcholine-enriched liquid disordered phase are freely accessible to AEA and their –OH group is fully available. For this reason, it can be assumed that AEA probably targets cholesterol in the Ld phase but not in the Lo phase. However, the main receptors for AEA (CB1 in the brain) are located in lipid raft domains [53,54]. Therefore, once AEA is inserted into the membrane, it probably remains bound to cholesterol which now acts as a membrane transport system for the neurotransmitter to reach its binding site in the Lo domains. 

## 6. Cholesterol-Dependent Anandamide Membrane Crossing

The interaction of AEA with plasma membrane cholesterol may also be associated with the mechanism of AEA transport through a biological membrane. Studies with model protein-free lipid bilayer systems indicate that AEA transport is strictly dependent on cholesterol which, above a threshold molar value (20% of total lipids), triggers the passage of AEA [34]. In this model, cholesterol is equally distributed in the two leaflets of the bilayer, which favors a biologically relevant tail-to-tail (mirror) arrangement [55,56]. According to this unique topology of cholesterol molecules, one can assume that AEA first binds to cholesterol in one leaflet and is then transmitted to the mirror cholesterol molecule in the other leaflet via a flip-flop mechanism [57]. Although performed with reconstituted membranes, these data demonstrate that AEA can be vectorially transported across a membrane by passive diffusion without the need for a dedicated transport protein. Nevertheless, the possibility that mirror cholesterol molecules could in some cases be associated with a transmembrane domain [58] of a receptor is discussed in Section 9. 

In a physiological context, AEA that crosses the membrane could either interact or not with a receptor such as CB1. The amount of AEA inserted in the pre-synaptic membrane is likely to exceed the number of available receptors. In any case, AEA will eventually leave the membrane and reach the cytoplasm where it will be captured by an intracellular transport system such as fatty acid binding proteins [59]. The final step will be the enzymatic degradation of AEA into arachidonic acid and ethanolamine by the fatty acyl amide hydrolase (FAAH) [60,61]. 

## 7. Endocannabinoid Binding to CB1 Receptors: In Silico Data

Several structural models of CB1 receptor based on class A (rhodopsin like) GPCR sequence homologies have been proposed [62]. Docking studies of endocannabinoid in CB1 receptor included in a phospholipid bilayer matrix showed that the binding domain is structured around four transmembrane helices (TMH), namely TMH2, TMH3, TMH6, and TMH7. When complexed to its receptor, AEA is locked in a constrained L-shape structure interacting with a hydrophobic pocket lined with a series of aromatic and branched amino acid residues (Phe, Leu, Val). Molecular dynamic simulations of the sixth TMH of CB1 (TMH6) in a phoshatidylcholine bilayer suggested that the first anchoring point of AEA is a groove containing the branched aliphatic residues Val-351 and Ile-354. Following this initial interaction, AEA is predicted to enter the receptor by interacting with a CWGP motif located between TMH6 and TMH7 [63]. Comparative docking studies of the CB1 receptor in complex with an agonist or with an antagonist described two conformational states correlated with two functionally distinct (active/inactive) states of the CB1 receptor, respectively. Biophysical studies indicated that the agonist-induced receptor activation is associated with a modification of the respective orientations of TMH3 and TMH6 which results in the separation of two aromatic residues, Phe-200 and Trp-356, which are initially in contact through a typical aromatic π−π interaction [62]. In contrast, both residues remain in the close vicinity of the antagonist-CB1 receptor complex which is considered the inactive state of the CB1 receptor. The computational results mentioned above describe accurately, at the Å scale, the details of the chemical interactions between endocannabinoids and their receptors. However, we suggest that these models could be refined to take into consideration the presence of cholesterol, which has been shown to be a key regulator of GPCR function [64,65]. In any case, all available models of endocannabinoid-CB1, agonist-CB1 and antagonist-CB1, highlight the conformational flexibility of the CB1 receptor binding site. In fact, the predicted ligand-dependent structural changes might reflect, at least in part, the relatively high mobility of the different TMHs. One could of course argue that it is precisely the absence of cholesterol in these in silico studies that may have artefactually increased the conformational flexibility of CB1.

## 8. G Protein-Coupled Receptors and CB1 Receptors: What Crystallographic Data Have Revealed 

The role of cholesterol in the stability and functions of GPCRs was raised around two decades ago [66,67]. However, the impact of cholesterol on GPCR structure remains to be fully clarified, chiefly because the successful crystallization of membrane proteins has long been impaired by the lack of appropriate methods [64]. Nowadays, the lipid cubic phase system has proved to be a useful approach to crystallize membrane proteins and in some cases, to co-crystallize membrane proteins with lipid assemblies [68,69]. The method is based on the use of monoolein, “a magic” lipid, which, in combination with a small amount of cholesterol, provides an appropriate environment for the concentration and subsequent crystallization of membrane proteins [70]. The use of crystallographic data to map functional cholesterol binding sites on various GPCRs has represented a breakthrough in our knowledge of the paradigm which governs the mode of association between cholesterol and integral membrane proteins belonging to the rhodopsin receptor-like superfamily [71]. A cholesterol consensus binding motif (CCM) was first defined by Hanson et al. for the human β2AR [72]. Unfortunately, the resolution of other cholesterol–GPCR crystal structures has revealed that the binding of cholesterol molecules at this CCM is not a universal rule [64]. Other cholesterol binding domains have been identified through computational and biophysical approaches and detected in various membrane proteins, including the large superfamily of GPCRs. These cholesterol binding motifs [73] are defined by linear consensus amino acid sequences such as (L/V)-X1-5-(Y)-X1-5-(K,R) (CRAC motif [74]), or its reverse form (K/R)-X1-5-(Y/F)-X1-5-(L,V) (CARC motif [75]). Both motifs are widely distributed over GPCRs, although there is no unique pattern of cholesterol binding to this family of receptors [64]. This variability relates to the number of cholesterol molecules bound to each receptor type, the TMH(s) of the receptor that display(s) these motifs, and their location in the outer and/or inner membrane leaflets [76]. 

In a number of instances, in silico and crystallization approaches converge. For the CB1 receptor, a cubic phase consisting of monoolein with 10% cholesterol allowed the crystallization of a CB1–cholesterol complex [77]. The cholesterol binding site of CB1 characterized by crystallization (Val-235→Ile-245) [77] partially overlapped with a CARC motif present in the inner leaflet of the plasma membrane (Lys-232→Ile-243) [58]. Interestingly, the presence of a surface-exposed tryptophan residue (Trp-241) within this motif accounted for more than 50% of the total energy of interaction between cholesterol and the fourth TMH of the CB1 receptor (as calculated from the atomic coordinates of the PDB file 5XRA [77]). 

There are two potential drawbacks to the lipid cubic phase crystallization system: (i) monoolein is actually not a membrane lipid; and (ii) the percentage of cholesterol generally used is 10%, whereas the average cholesterol content of a real membrane is around 30%. One could therefore ask what would happen if a higher amount of cholesterol was used and if monoolein was substituted by a more biologically relevant membrane lipid such as phosphatidylcholine (POPC). A partial answer to this question was provided in a study of cholesterol dynamic exchange with β2AR in a lipid cubic phase using fluorescence and nuclear magnetic resonance (NMR) spectroscopy methods [78]. A high affinity cooperativity binding (with sub-nanomolar affinity constant value) was associated with slow exchanges of cholesterol in the minute time-scale. Conversely, low affinity cholesterol binding sites, corresponding to fast exchanges of cholesterol in the second time-scale, have been detected using saturation transfer difference by NMR spectroscopy. The formal identification of two pools of cholesterol associated with β2AR allowed a functional discrimination between tightly bound cholesterol (non-annular cholesterol) and low affinity bound cholesterol (annular cholesterol). The tightly bound cholesterol stabilizes the three-dimensional (3D) structure of the GPCR in the membrane environment, thereby creating sufficiently stable conditions to allow surrounding cholesterol to interact with receptors through exchangeable, low affinity binding sites. 

A complementary in silico approach has been developed for identifying cholesterol binding motifs in the β2AR [79]. In this case, extensive molecular dynamic simulations of the receptor in the microsecond range have been carried out in POPC/cholesterol bilayer models. These studies confirmed that cholesterol increases the compactness of the receptor structure and enhances its conformational stability in both the active and inactive states. Most importantly, these data strongly suggest the existence of two distinct pools of cholesterol bound to eight potential cholesterol binding sites: three high cholesterol affinity binding sites and five low cholesterol binding sites corresponding to long lifetime binding (µs time scale) and short lifetime binding (10–100 ns timescale), respectively. The latter cholesterol type would probably not appear in crystal structures but its contribution to the β2AR structure compactness and stability remains important.

**Figure 7:**
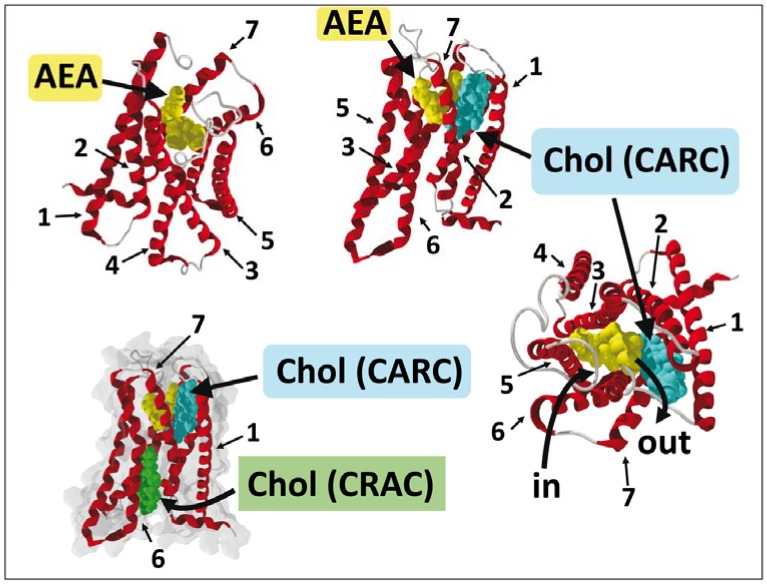
Docking of AEA in the CB1 receptor. Several views of the AEA/CB1 complex, with and without cholesterol (Chol) are shown. The transmembrane helices (TMH) domains (numbered from 1 to 7) of the CB1 receptor (retrieved from PDB file 5XRA) are in red. Anandamide (in yellow) was docked in the ligand binding pocket. Note the typical L-shape conformation of AEA bound to the receptor (upper left panel). Two cholesterol molecules bound to the seventh TMH in a typical CARC/CRAC mirror position are shown in the lower left panel (cholesterol bound to CARC is in cyan, the one bound to CRAC is in green). The entry (in) and exit (out) pathways of AEA are indicated (right panel). Molecular docking was performed by J. Fantini according to the method described in reference [58].

Recently, crystal structures of two agonist (AM11542 and AM841)-bound human CB1 receptors have been solved at 2.8 and 2.95 Å resolution, respectively [77]. A structure of the receptor in complex with an antagonist (AM6538) was previously published by the same team [80]. Overall, these results emphasized the plasticity of the CB1 binding pocket which can fit to diverse arrays of ligands with variable sizes and shapes. From the crystal structure of the agonist-bound human cannabinoid CB1 receptor [77], we have built a model of the cannabinoid receptor binding domain associated with AEA (Figure 7). A similar modeling exercise has been performed by Hua et al. [77] and by Sabatucci et al. [81]. The AEA binding site has been localized in a crevasse constituted by three transmembrane helices (TMH3, TMH6 and TMH7) that together form a closed bundle in the membrane space. Consistent with previous in silico studies [62,63], AEA adopts a typical L-shape as soon as it reaches its binding pocket. Interestingly, this L-shape is clearly distinct from the three main types of anandamide conformers (hairpin, linear or snake-like) that can be characterized by molecular dynamics studies (Figure 2). Therefore, it appears that the binding pocket of CB1 may act as a mould able to temporarily stabilize a constrained conformer of AEA. In fact, this situation is particularly favorable to ensure the rapid release of AEA from CB1. The last issue that remains to be resolved concerns the possible role of cholesterol in the formation and the disruption of CB1/AEA complexes.

## 9. A Dual Cholesterol–CB1 Receptor Model for Anandamide

Considering the high level of sequence and structural homology between the β2AR and other rhodopsin-like GPCRs, we can reasonably assume that the cholesterol distribution around the β2AR [78] can be generalized to cover most members of the GPCRs superfamily. In this case, annular cholesterol molecules surrounding CB1 receptors attract AEA/cholesterol complexes that would otherwise diffuse stochastically in the membrane. From the different models available in the literature, one can evaluate the affinity of AEA for cholesterol and for the CB1 receptor. The docking of AEA onto the ligand-binding pocket of CB1 (Figure 7) results in a high affinity complex with a predicted energy of interaction of −136 kJ·mol^−1^. In contrast, the energy of interaction of an AEA/cholesterol complex in the membrane environment has been estimated to be only −30.3 kJ·mol^−1^ [23]. According to these estimations, it is clear that AEA has a much higher affinity for CB1 than for cholesterol. Therefore, it can be postulated that when an AEA/cholesterol complex meets CB1, AEA would leave cholesterol to move into the ligand binding site. 

The entrance of AEA has been localized between TMH6 and TMH7, in a region definitely located in the exofacial leaflet of the plasma membrane (Figure 7). Interestingly, TMH7 displays two potential cholesterol binding domains, one in each leaflet of the membrane, in the typical “mirror” topology that we have described [58]. In the outer leaflet, there is a CARC domain [75] onto which cholesterol may bind just in front of the AEA binding pocket (Figure 7). There is very little overlap between the amino acid residues that constitute the AEA binding pocket and this CARC domain and, hence, CB1 may perfectly bind both AEA and cholesterol simultaneously. The position of this particular CARC motif might of course be totally coincidental. However, it is tempting to speculate that cholesterol could in fact interact with this CARC domain to attract AEA from its binding pocket and extrude it from the receptor. In this case, cholesterol would facilitate the relaxation of the receptor by regenerating its unbound state. The CRAC motif [74] of TMH7 is located in the cytoplasmic (inner) leaflet of the membrane (Figure 7). Accordingly, it cannot be directly involved in the AEA binding/release process which takes place in the exofacial (outer) membrane leaflet. Once again, the location of this CRAC domain might be purely coincidental. However, a closer view of the model shown in Figure 7 suggests that the extraction of AEA by cholesterol (via the CARC motif of the outer leaflet) would probably induce a slight opening of the channel between TMH7 and TMH1, which might therefore act as an exit channel (Figure 7). Under these circumstances, the cholesterol molecule bound to the CRAC domain (in the inner leaflet) would help stabilize the 3D structure of CB1, thereby preventing its collapse. Moreover, it could also attract the AEA molecules that leave the receptor at the level of the CARC exit domain in the inner leaflet, a possibility consistent with the observation that cholesterol stimulates the transmembrane transport of cholesterol in reconstituted bilayer systems [34]. Neither the CARC nor the CRAC domain would bind cholesterol in the absence of AEA, because of the “annular/low affinity” nature of both sites. Finally, if cholesterol facilitates the exit of AEA from CB1, a distinct mechanism takes place when AEA enters its binding site. In fact, the ligand binding pocket of CB1 does not display any CARC, CRAC, or other cholesterol binding motif. Therefore, it can be assumed that once that the AEA/cholesterol complex is destroyed, AEA moves inside the binding pocket whereas cholesterol may interact with either CARC or CRAC in TMH7. This scenario supposes that the entry and exit of AEA are distinct phenomena that occur at distant sites of the receptor, as previously shown for rhodopsin, a receptor which also interacts with a lipid ligand [82,83]. Future studies will be necessary to assess whether such a mechanism applies for AEA and CB1, that is, whether AEA follows a unidirectional path to enter, interact with, and finally leave the receptor through a coordinated cholesterol-dependent process. If this were the case, it would explain for the first time the striking occurrence of the dual CARC/CRAC motif within the same TMH.

## 10. Receptor-Independent Signaling of Anandamide

Anandamide belongs to the family of brain bioactive lipids which includes several important signaling molecules such as sphingomyelin or ganglioside GM1 [84]. As a ubiquitous lipid widely distributed in both central and peripheral tissues, AEA regulates a broad range of physiological activities including neurotransmission, cardiovascular, respiratory, and food intake functions [15]. Anandamide also controls important biological processes associated with cellular proliferation, survival, or programed cell death [85].

It is generally accepted that AEA exerts paracrine effects mediated by cannabinoid receptors (CB1 or CB2). However, it has been shown that AEA may also act independently of cannabinoid receptors through selective interactions with membrane lipids. In particular, several AEA functions seem to involve raft lipids, including cholesterol [86,87,88,89,90]. In a previous work, we have shown that cholesterol could mediate the insertion and transport of AEA in a synthetic membrane model [34]. In addition to membrane cholesterol, this transport requires a cytoplasmic protein such as the fatty acid binding protein that could bind AEA as soon as it leaves the membrane environment [9,34,39,59,91,92,93].

Ceramides have also been shown to bind AEA when present in the plasma membrane [50]. The human neuroblastoma cell line SH-SY5Y has been used to unravel the role played by ceramides in the biological activity of AEA [50]. Ceramides are stress lipids involved in various signaling pathways leading to apoptosis or growth arrest [94]. Following the enzymatic hydrolysis of sphingomyelin by an exogenous addition of sphingomyelinase to neural SH-SY5Y cells in culture, ceramides appear transiently and return quickly to their basal levels. The presence of ceramides has a marked impact on the stability of AEA. In control cells incubated with AEA alone, the lipid neurotransmitter is readily hydrolyzed by a fatty acid amide hydrolase (FAAH). In contrast, when the cells are pre-treated with sphingomyelinase prior to AEA, we observed that AEA is preserved from enzymatic hydrolysis. Interestingly, a similar stabilization of ceramide content was also evidenced, indicating that the simultaneous presence of AEA and ceramides blocks their respective degradation pathways. In other words, the accumulation of both ceramides and AEA might indicate that each of these lipids has a mutual inhibitory effect on the metabolism of the other. Molecular dynamic simulations of the formation of AEA/lipid complexes in a POPC matrix made it possible to estimate the energy of interaction of the AEA/cholesterol complex to −30 kJ·mol^−1^. The interaction of cholesterol with 2-AG, as determined by molecular docking approaches, is of the same order (−33 kJ·mol^−1^). In the case of ceramide, the complex with AEA was significantly more efficient, reaching −41 kJ·mol^−1^ for one AEA bound to one ceramide (binary complex), and −90 kJ·mol^−1^ for one AEA interacting with two ceramide molecules (ternary complex). Another interesting feature of AEA/lipid complexes is the regulation of the accessibility of AEA to FAAH. When AEA is bound to cholesterol, its polar head group emerges from the membrane and is thus fully accessible to enzymatic hydrolysis. In contrast, when AEA is bound to ceramide (either in a binary or in a ternary complex), its polar head group is totally masked by ceramide, so that the complex, dipped in the membrane, is not accessible to FAAH. The hydration of these complexes around the polar head groups of those AEA/lipid complexes also matters for regulating the accessibility of AEA to FAAH. In silico calculations have shown that 48 molecules of water can be associated with a binary AEA/ceramide complex whereas only 15 water molecules may interact with an AEA/cholesterol complex. Structural data combined with docking studies [92] have suggested that a salt bridge between Arg-486 and Asp-403 in FAAH may prevent the access of AEA to the active site of the enzyme. In fact, the hydrolysis reaction requires that this salt bridge is broken by a conformational change triggered by cholesterol, a process that can be interpreted as a specific case of interfacial activation [95,96]. Our in silico data suggest that the highly hydrated AEA/ceramides complexes cannot induce the interfacial activation of FAAH [50,23]. 

In another serie of studies we characterized the effect of AEA/ceramide complexes on ceramide metabolism. The physiological pathway leading to ceramide inactivation involves enzymatic hydrolysis which produces one sphingosine and one saturated fatty acid from each ceramide molecule [97]. When SH-SY5Y cells were treated with sphingomyelinase, the immediate effect was an increase in ceramide content rapidly followed by the transient appearance of both degradation products. In contrast, when the cells were first treated with sphingomyelinase and then incubated with AEA, ceramides were detected for a longer time, suggesting that when ceramide is entrapped in a complex with AEA, its metabolism is significantly delayed. The molecular mechanism responsible for this effect may also be controlled by the hydration level of the AEA/ceramide complex which could limit the accessibility of ceramide to ceramidase. In other words, one can propose a general effect of hydration of AEA/lipid complexes which might be responsible for an inhibition of lipid substrate anchorage to hydrolysis enzymes (FAAH for AEA and ceramidase for ceramides). This inhibitory effect has to be both subtle and specific. An interesting aspect of ceramidase activity is that it operates on small ceramide clusters [98]. With this in mind, how can the enzyme make a clear-cut distinction between a ceramide molecule complexed with another ceramide molecule (functional substrate for ceramidase) and ceramide combined with AEA (not recognized by ceramidase)? This issue is made even more complex by the fact that our molecular modeling simulations showed that the association between two molecules of ceramide (Gibbs free energy, ∆G = −79.5 kJ·mol^−1^) is stronger than the AEA/ceramide association (∆G = −45 kJ·mol^−1^). However, the real substrate of ceramidase is not a cluster of ceramide molecules but a hydrated cluster of ceramide, which makes a significant difference. Indeed, ceramide dimers are hydrated by only 18 water molecules whereas an AEA/ceramide complex interacts with 48 molecules of water [50,23]. Overall, these data suggest that the functional anchorage of a lipid substrate to its hydrolyzing enzyme is controlled by two distinct parameters: (i) its degree of hydration; and (ii) its association with surrounding lipids that can either prevent or facilitate the formation of an enzyme–substrate complex. These parameters may be considered in the context of the lateral organization of membrane lipids that can be topologically described with two distinct theoretical models [99]: (i) the super-lattice model and (ii) the segregated arrangement model. In the latter case, ceramide molecules are clustered into specific domains with a low level of hydration favoring the action of ceramidase. In the super-lattice model, ceramide is homogeneously distributed in the membrane, favoring its interaction with AEA and thus preventing its enzymatic hydrolysis.

Another consequence of sphingomyelinase treatment is the production of intracellular vesicles that are enriched in ceramides which aggregate into specific clusters according to the segregated arrangement model. In this case, the reduced hydration surface associated with packing defects at the boundaries of these micro-domains [100] would favor ceramidase anchoring and ceramide hydrolysis. This process could be functionally impaired if the cells are submitted to an AEA stimulation at the same time, suggesting that AEA might exert a fine control of ceramide signaling. 

Finally, ceramides have been shown to induce a negative membrane curvature associated with the formation of endocytic vesicles [101,102]. In the presence of AEA, the formation of AEA/ceramide complexes could suppress this effect, allowing the cells to efficiently repair the membrane damage triggered by sphingomyelinase treatments. 

## 11. How Can We Discriminate between Receptor-Dependent and Receptor-Independent Effects of Anandamide?

**Figure 8:**
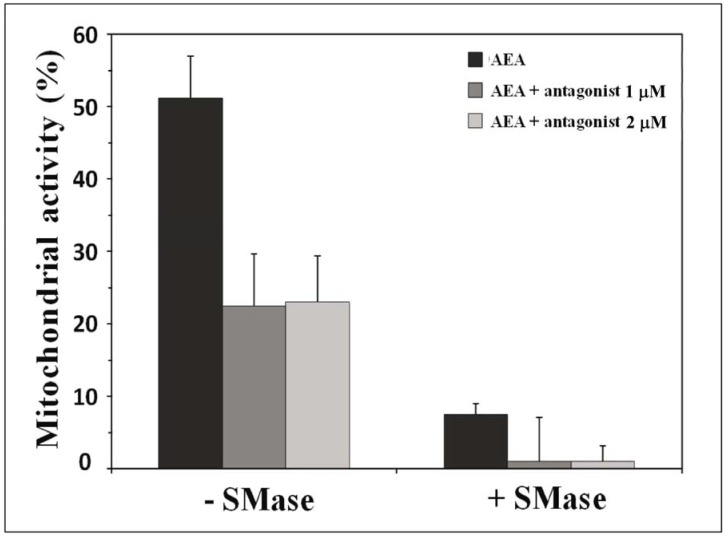
CB1-independent effects of AEA revealed by a CB1 antagonist. SH-SY5Y cells were incubated with (right panel) or without neutral sphingomyelinase (left panel), then treated during 24 h with an AEA concentration which reduced mitochondrial activity by half, and different concentrations of antagonist (1 µM or 2 µM). The mitochondrial activity was determined by the (3-(4,5-dimethylthiazol-2-yl)-5-(3-carboxymethoxyphenyl)-2-(4-sulfophenyl)-2H-tetrazolium) MTS test. The results are means ± standard deviation (SD) of three independent experiments [105]. Abbreviations: −SMase, without sphingomyelinase; +SMase, plus sphingomyelinase.

It is generally accepted that CB1 receptors constitute the main target of AEA [13]. However, it has also been shown that some AEA effects can occur through receptor-independent mechanisms [103], possibly involving plasma membrane lipids [90]. Our data identified cholesterol as a target for AEA in the plasma membrane of neural cells [34]. We have used the AEA antagonist SR141716A (rimonabant [104]) to determine the involvement of CB1 in the cytotoxicity induced by AEA. In these experiments, SH-SY5Y cells were incubated with AEA in the presence of increasing concentrations of the antagonist and mitochondrial cytotoxicity was measured by the (3-(4,5-dimethylthiazol-2-yl)-5-(3-carboxymethoxyphenyl)-2-(4-sulfophenyl)-2H-tetrazolium) (MTS) assay [50]. In agreement with previous studies [90], rimonabant did not protect the cells against the cytotoxic effects of AEA (Figure 8). Pre-incubating the cells with sphingomyelinase resulted in an increased toxicity of AEA. This hypersensitization of cytotoxicity could be attributed to an increase in cellular ceramide content since it was mimicked by an exogenous addition of C2-ceramide [50]. Once again, the antagonist did not protect the cells from AEA toxicity in sphingomyelinase-treated cells exhibiting a high level of ceramides (Figure 8). 

Taken together, these data suggest that: (i) the cytotoxicity induced by AEA is a CB1-independent process; and (ii) such cytotoxic effects of AEA are hypersensitized by ceramide, also through a CB1-independent pathway. It could be argued that these CB1-independent mechanisms involve one or several of the receptors known to be used by AEA in addition to CB1 [106,107]. The fact that the effects are modulated by ceramide could, at least, reflect a higher or lower sensitivity of the cell system depending on the ceramide tone of the system. 

**Figure 9:**
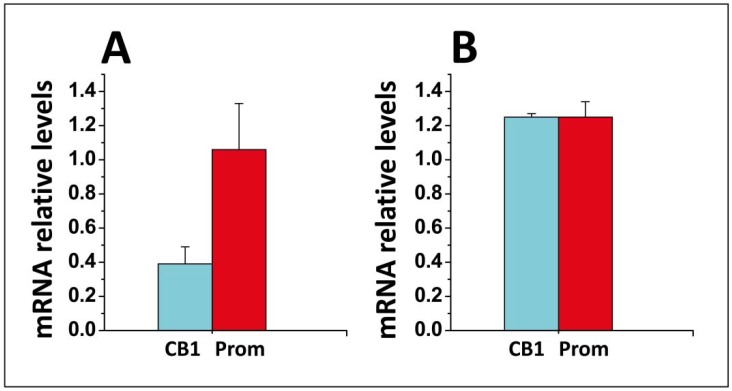
Real-time polymerase chain reaction (PCR) of CB1 messenger RNAs (mRNAs) in SH-SY5Y cells. **A:** Effect of a 24 h AEA treatment on the relative expression of CB1 and prominin (Prom) mRNAs. The results are expressed as the fold increase measured in anandamide-treated cells vs., control untreated cells (mean ± standard deviation (SD), *n* = 2). The data were presented as the fold change (2^−ΔΔCt^) in gene expression normalized to an endogenous reference gene (glyceraldehyde 3-phosphate dehydrogenase, GAPDH) and relative to a calibrator (non-treated cells). The quantification revealed that AEA induced a 2.5-fold decrease in CB1 gene expression whereas prominin gene expression was only slightly affected (1.06-fold increase). **B:** The data show the effect of anandamide on sphingomyelinase-treated cells. In this case, both CB1 and prominin gene expression were slightly higher (1.25-fold) by AEA [108].

In parallel experiments, the level of expression of CB1 messenger RNAs (mRNAs) was determined by real-time polymerase chain reaction (qPCR) with glyceraldehyde 3-phosphate dehydrogenase (GAPDH) mRNAs used as an endogenous control and prominin mRNAs as independent controls. The data indicated that AEA induced a specific and dramatic decrease in CB1 mRNAs whereas it did not affect prominin mRNAs (Figure 9). This feedback regulation of CB1 expression was found to be suppressed by ceramide, probably because this lipid forms a tight complex with AEA in the membrane environment. This AEA/ceramide complex is more cytotoxic than AEA alone, but its toxic effects are not mediated by CB1, probably because the interaction with ceramide prevents the binding of AEA to its receptor. 

We have now deciphered several key aspects of the fundaments of the molecular mechanisms controlling insertion of AEA in the plasma membrane of receptive cells expressing CB1 receptors. These mechanisms are clearly linked to the lipid nature of AEA and its solubility properties which are not consistent with the classical pathway leading to the activation of synaptic receptors whose ligand binding site is directly accessible from the extracellular space. Among all membrane lipids, AEA displays an unexpected selectivity for cholesterol and ceramides, both of which function as critical regulators of AEA biological activity. Cholesterol ensures the membrane insertion of AEA and its transport to either CB1 receptors (signal transduction pathway) and/or to intracellular proteins (hydrolysis pathway). In contrast with cholesterol, ceramides are present in the membrane only after the activation of sphingomyelinase through a specific signaling mechanism. It is also important to note that in addition to CB1, AEA may interact with other membrane proteins [109] such as L-type Ca^2+^ channels [110], the ionotropic serotonin receptor 5-HT3 [111], or the vanilloid receptor subtype 1 (TRPV1) [106]. Interestingly, cholesterol has been shown to regulate the function of all these proteins [112,113,114]. Ceramide may also display some regulatory activity on L-type Ca^2+^ currents [115] but—to the best of our knowledge—not on the other AEA receptors, including CB1. From a clinical point of view, the exacerbated toxicity of AEA in the presence of ceramide [50] and the striking interplay between the two signaling systems [116] should be taken into consideration for developing new anti-tumoral strategies based on the use of endocannabinoids [50,117,118]. In this respect, understanding the molecular mechanisms underlying the biological activity of AEA and related endocannabinoids is of primary importance for the use of these molecules in the treatment of human diseases. We hope that the key features of AEA described and discussed in the present review will stimulate a fruitful exchange of ideas and promote original and rational clinical applications.
